# Changes in activity of metabolic and regulatory pathways during germination of *S. coelicolor*

**DOI:** 10.1186/1471-2164-15-1173

**Published:** 2014-12-23

**Authors:** Jan Bobek, Eva Strakova, Alice Zikova, Jiri Vohradsky

**Affiliations:** Institute of Microbiology, Academy of Sciences of the Czech Republic, Laboratory of Bioinformatics, Vídeňská 1083, 142 20 Prague 4, Czech Republic; Institute of Immunology and Microbiology, First Faculty of Medicine, Charles University in Prague, Studničkova 7, 128 00 Praha 2, Czech Republic; Chemistry Department, Faculty of Science, J. E. Purkinje University, 400 96 Ústí nad Labem, Czech Republic

**Keywords:** Germination, *Streptomyces*, Microarrays, Sigma factors, Metabolic pathways

## Abstract

**Background:**

Bacterial spore germination is a developmental process during which all required metabolic pathways are restored to transfer cells from their dormant state into vegetative growth. *Streptomyces* are soil dwelling filamentous bacteria with complex life cycle, studied mostly for they ability to synthesize secondary metabolites including antibiotics.

**Results:**

Here, we present a systematic approach that analyzes gene expression data obtained from 13 time points taken over 5.5 h of *Streptomyces* germination. Genes whose expression was significantly enhanced/diminished during the time-course were identified, and classified to metabolic and regulatory pathways. The classification into metabolic pathways revealed timing of the activation of specific pathways during the course of germination. The analysis also identified remarkable changes in the expression of specific sigma factors over the course of germination. Based on our knowledge of the targets of these factors, we speculate on their possible roles during germination. Among the factors whose expression was enhanced during the initial part of germination, SigE is though to manage cell wall reconstruction, SigR controls protein re-aggregation, and others (SigH, SigB, SigI, SigJ) control osmotic and oxidative stress responses.

**Conclusions:**

From the results, we conclude that most of the metabolic pathway mRNAs required for the initial phases of germination were synthesized during the sporulation process and stably conserved in the spore. After rehydration in growth medium, the stored mRNAs are being degraded and resynthesized during first hour. From the analysis of sigma factors we conclude that conditions favoring germination evoke stress-like cell responses.

**Electronic supplementary material:**

The online version of this article (doi:10.1186/1471-2164-15-1173) contains supplementary material, which is available to authorized users.

## Background

A prokaryotic spore is a type of resting cell whose function is mainly to guard the genetic information of an organism during unfavorable conditions and to help its spreading. Spore germination is the subsequent developmental step that transfers these cells from their dormant state into metabolically active vegetative growth. How the metabolically inactive dormant spore is adapted for eventual germination, how cell metabolism is reactivated and how the cell is differentiated during this process are the questions that attract our attention. The genus *Streptomyces* is used here as a model organism. Streptomycetes are important producers of clinically important antibiotics, and their antibiotic production is an object of intensive scientific interest [1]. The complex cell differentiation of streptomycetes comprises the formation of vegetative substrate mycelium, aerial mycelium and sporulation [2–4]. In the model species *S. coelicolor*, spore formation is preceded by twisting of the aerial hyphae, followed by a synchronized cell division of the hyphae, leading to the formation of haploid compartments. These compartments assemble an envelope layer with a hydrophobic external sheath. The coat is composed from chaplin and rodlin hydrophobic proteins that form a basket-like structure, for more details see review [3]. The resulting spores then undergo maturation, during which their water content is decreased and a grey pigment is produced. Although streptomycetes spores are quite resistant to desiccation, their spores are less resistant to other environmental extremes than endospores of *Clostridia* or *Bacilli*. Therefore, they can rather be seen as dispersal agents [5].

Whereas sporulation and antibiotic production are well studied, much less attention has been paid to germination. Spores are re-activated from dormancy in an aqueous medium. Spores lose their hydrophobicity, which leads to water influx and thus swelling. Germination may be stimulated by heat shock [6] or by mechanical incidences that disrupt the external sheath [7–9].

The development of dormant spores into vegetative forms is a sequential process. First, hydrolytic enzymes such as RpfA and SwlA degrade cell wall peptidoglycans [10]. After 60 min of incubation, intensive ribosome synthesis occurs [11]. This phase is characterized by enhanced rates of RNA and protein synthesis and the first DNA replication occurs between 120 – 150 min. Then germination tubes rise through the outer spore wall and vegetative hyphae start branching [12, 13]. Previous studies of *Streptomyces* development including germination [14–17] mostly relied on proteomics [14–17] or the combination of proteomics and transcriptomics [14, 18, 19] approaches.

Genome wide analytic methods, such as microarrays or transcriptome sequencing followed by biocomputational modeling [20, 21] offers a global view of gene/protein expression guiding the characterization of complex regulatory networks. Expression data obtained from these experiments are then used to estimate the levels of individual proteins. However, specifically with respect to germination, we are as yet unable to distinguish which expression levels of different mRNAs, identified by microarrays, are critical for the appropriate functioning of their protein products. In the present study, microarray data were processed to determine how and at which stage of germination gene expression changes become significant. This approach provides new details that contribute to our understanding of the germination process on a global scale.

In order to have a view on the gene expression dynamics of the different genes specifically expressed in the course of the germination process, we collected RNA samples every 30-min from dormant spores and up to 5.5 h of growth after heat shock (a total of 13 time points) from at least three biological replicates.

## Results and discussion

The aim of this work was to identify genes that are differentially expressed between two consecutive time points during the germination of *S. coelicolor*. Analyzing differential expression allowed us to identify genes and, consequently, metabolic and regulatory pathways whose expressions were enhanced or diminished between the two time points.

Throughout the paper, all references to the changes in gene expression levels concern the ratio between expression levels in time point t_j+1_ and t_j_ (periods marked as t1-t2, t2-t3 etc., see paragraph Differential expression analysis in Methods). The terms used are usually: “enhanced/diminished expression”, or “up/down regulation”, or “activation/deactivation”. These terms have no relation to actual molecular mechanism that led to the changes in expression levels of a particular gene, but refer solely to the above mentioned expression levels ratios. By determining the genes with enhanced/diminished expression, we can infer changes in the corresponding pathway map over the observed germination period and correlate these changes with morphological and physiological development.

**Figure 1:**
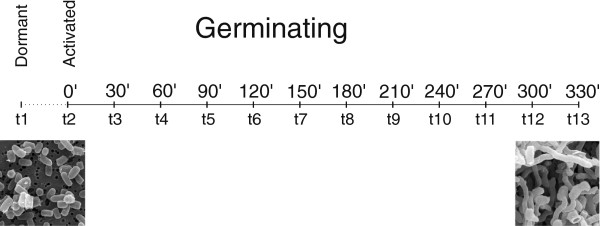
**Relationship between expression intervals (t) and sample collection times.**

Germination was monitored from dormant state of spores up to 5.5 h of growth after heat spore activation, and RNA samples were collected at 30-min intervals from at least three biological replicates (Figure 1). The sample set contained data from 13 time points, including dormant and activated spores. The signals from microarray spots corresponding to individual genes were arranged in a dataset for further processing. Genes whose expression was enhanced or diminished between two consecutive time points were identified by t-test for equality of means, and genes that exhibited significant change were checked for the fold change. Those genes, whose expression changed by more than 2-fold, were selected (Additional file 1). Altogether, increased abundance was observed for 1958 individual genes at least once between two consecutive time points, and decreased abundance was observed for 1960 genes. Almost one third (639) of the genes in the enhanced set and 687 genes in the diminished set were classified as “Unknown” or “Not classified” (according to the Sanger *S. coelicolor* genome sequence database annotation), and another 237 genes in the enhanced set and 275 in the diminished set were classified as hypothetical. In order to identify the metabolic pathways in which the identified genes were involved, the KEGG (http://www.genome.jp/kegg/pathway.html) database of *S. coelicolor* genes and their pathway ontologies was downloaded [22]. For *S. coelicolor*, the KEGG database records 1369 individual genes assigned to 118 pathways and 19 functional groups (Amino acid metabolism, Biosynthesis of other secondary metabolites, Carbohydrate metabolism, TCA cycle/pentose phosphate/glycolysis, Cell motility, Energy metabolism, Folding, sorting and degradation, Glycan biosynthesis and metabolism, Lipid metabolism, Membrane transport, Metabolism of cofactors and vitamins, Metabolism of other amino acids, Metabolism of terpenoids and polyketides, Nucleotide metabolism, Replication and repair, Signal transduction, Transcription, Translation, Xenobiotics biodegradation and metabolism). The groups “Biosynthesis of other secondary metabolites”, “Cell motility”, and “Glycan biosynthesis and metabolism” were excluded from this set, as the expression of less than 3 genes was significantly changed. Distribution of the genes to individual metabolic groups followed the scheme given by KEGG and will be used throughout the paper. Altogether, 461 individual genes that were induced and 385 that were repressed at least once between two consecutive time points throughout the whole observed period were identified among the genes recorded in KEGG. This set was complemented with 45 sigma factors whose analysis is discussed further below.

**Figure 2:**
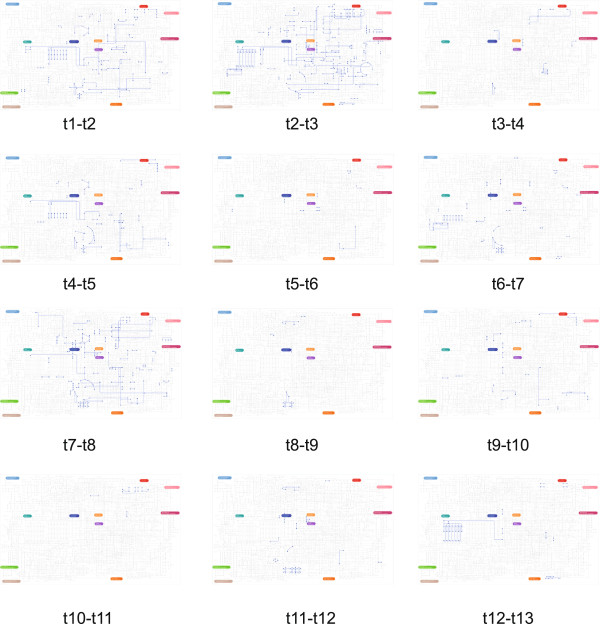
**Pathway maps with highlighted genes and pathways activated at individual time intervals.**

**Figure 3:**
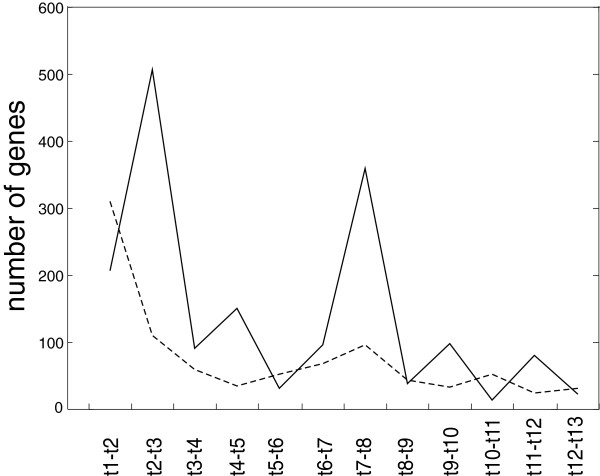
**Total number of genes with enhanced and diminished (dashed) expression between two consecutive time points.**

The genes with enhanced expression in each time interval were positioned on the KEGG pathway base map. The pathway diagrams with the genes with enhanced expression highlighted in the KEGG pathway base map is shown in Figure 2, and a movie representing the temporal transition of the incident pathways is available in additonal file 2. The total number of enhanced/diminished genes between two individual time points is shown in Figure 3.

**Figure 4:**
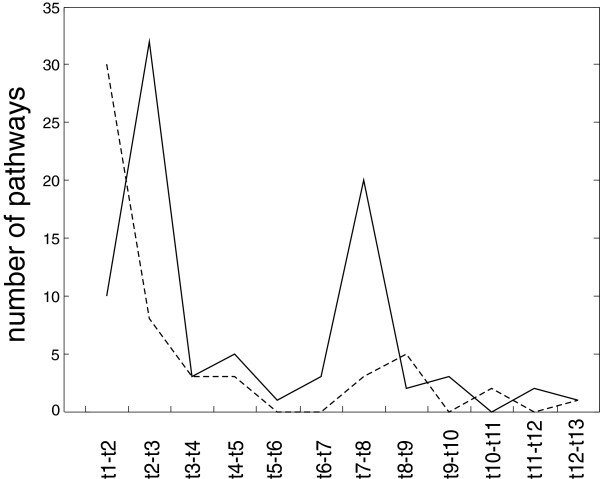
**Number of pathways with >3 genes whose expression was enhanced/diminished (dashed) between two consecutive time intervals.**

Most of the genes were differentially induced between points t2-t3 (0–30 min) and t7-t8 (150–180 min). By contrast, most of the diminished genes were detected at the onset of germination between time points t1 and t2. In practical terms, the highest decrease in individual gene expression occurring between t1-t2 indicated that a considerable mRNA pool persisted in the dormant spores and was degraded during their activation (the persistency of mRNA pool in dormant spores was evidenced in [23]. The number of pathways that were enhanced/diminished during the individual time intervals is shown in Figure 4.

**Figure 5:**
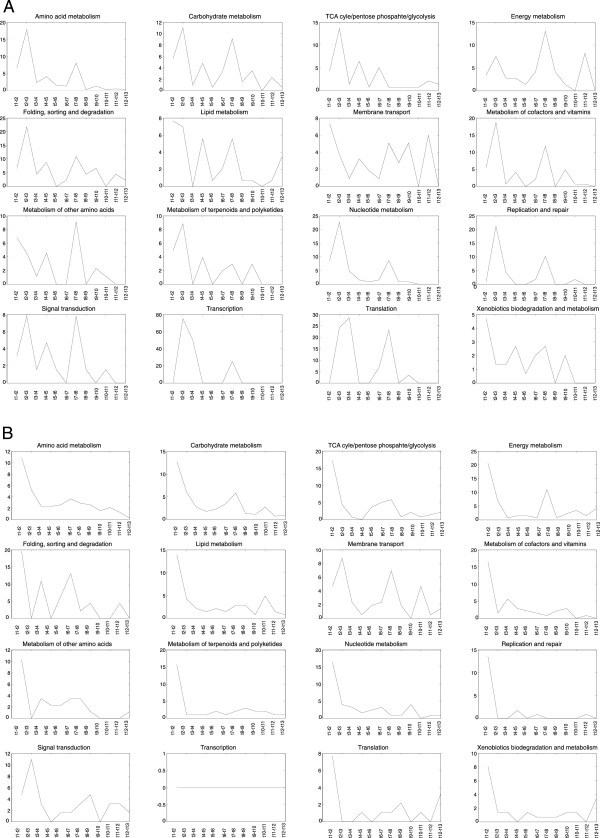
**Percentage of the number of genes with enhanced (A) or diminished (B) expression as assigned to individual functional groups.** The percentage is related to the total number of genes in a given metabolic group.

As in Figure 3, it is apparent that there are two time intervals during which substantial numbers of pathways were activated (t2-t3 and t7-t8). In between these time points, metabolic activity was constant; expression of the genes in the active pathways was neither enhanced nor diminished, within the experimental error limits. Most pathways were diminished in the first time interval, during which the levels for most of the messengers of the incident pathways were declining. Subsequently, the number of pathways down regulated remained on the level of the experimental error. The number of enhanced/diminished genes assigned to individual functional groups is shown in Figure 5.

The profiles in Figure 5A follow two trends – the first and largest copied the course of the whole set (Figure 3), and the second exhibited a maximum in the first time interval (t1-t2) (lipid metabolism, membrane transport, metabolism of other amino acids and genes of xenobiotic metabolism); in other time points, the numbers of genes with enhanced expression randomly fluctuated.

### Individual functional groups

The list of metabolic pathways and individual functional groups whose gene expression was increased/diminished during different periods of germination is given in Table 1.

**Table 1:** **List of metabolic pathways and functional groups whose genes expression was increased/diminished during different periods of germination**

Functional group	Deactivation in t1-t2 spore rehydration	Activation in t2-t3	Activation in t7-t8 first DNA replication	Other periods	Note
**Amino acid metabolism pathways**	250 Alanine, aspartate and glutamate		250 Alanine, aspartate and glutamate		
	260 Glycine, serine and threonine		260 Glycine, serine and threonine		
	270 Cysteine and methionine		270 Cysteine and methionine		
	280 Valine, leucine and isoleucine degradation		280 Valine, leucine and isoleucine degradation		
	290 Valine, leucine and isoleucine biosynthesis		290 Valine, leucine and isoleucine biosynthesis		
	300 Lysine biosynthesis		300 Lysine biosynthesis		
	310 Lysine degradation		330 Arginine and proline		
	330 Arginine and proline		340 Histidine400 Phenylalanine, tyrosine and tryptophan biosynthesis		
	340 Histidine				
	350 Tyrosine 360 Phenylalanine				
	380 Tryptophan				
	400 Phenylalanine, tyrosine and tryptophan biosynthesis				
**Carbohydrate metabolism**	TCA cycle/pentose phosphate/glycolysis/gluconeogenesis	TCA cycle/pentose phosphate/glycolysis pathways	TCA cycle-associated pathways and genes.	Deactivated in t7-t8	the majority - 23 of 57 were associated with the TCA cycle/pentose phosphate/glycolysis pathways genes
	40 Pentose and glucuronate interconversions	630 Glyoxylate and dicarboxylate metabolism	40 Pentose and glucuronate interconversions (2)	10 of 30 of the TCA cycle-associated pathways	
	51 Fructose and mannose metabolism	640 Propanoate metabolism	51 Fructose and mannose metabolism	52 Galactose metabolism	
	500 Starch and sucrose metabolism	650 Butanoate metabolism	500 Starch and sucrose metabolism	500 Starch and sucrose metabolism	
	520 Amino sugar and nucleotide sugar metabolism	660 C5-Branched dibasic acid metabolism.	52 Galactose metabolism	520 Amino sugar and nucleotide sugar metabolism	
	562 Inositol phosphate metabolism		53 Ascorbate and aldarate metabolism	562 Inositol phosphate metabolism	
	630 Glyoxylate and dicarboxylate metabolism		520 Amino sugar and nucleotide sugar metabolism	650 Butanoate metabolism	
	650 Butanoate metabolism.		562 Inositol phosphate metabolism	660 C5-Branched dibasic acid metabolism	
			630 Glyoxylate and dicarboxylate metabolism		
			640 Propanoate metabolism		
			650 Butanoate metabolism		
**Energy metabolism**	190 Oxidative phosphorylation,	190 Oxidative phosphorylation	190 Oxidative phosphorylatio	genes associated with cytochrome C activated in t11-t12	A minor portion of the genes with diminished expression at t7-t8 only, represented catalase (catA and B; SCO379 and 0666) and nitrate and nitrite reductases (SCO4947, 6102 and 6532)
	680 Methane	680 Methane metabolism,	680 methane metabolism		
	910 Nitrogen metabolisms	910 Nitrogen metabolism,	910 nitrogen metabolism		
**Folding, sorting and degradation**	20S Proteasome subunits (SCO1643 and 1644) and a Signal peptidase I (SCO5597 and 5598).	3018 RNA degradation	modest activation of chaperone GroEL (SCO4296 and 4762)	DnaK and GroEL was for both diminished at t6-t7.	
		3060 Protein export			
**Lipid metabolism and membrane transport**	Acetyl CoA carboxylase and acetyltransferase (SCO2445, 5399)			activation at (t1-t2) –	
	561 Glycerolipid metabolism			61 and 71 Fatty acid biosynthesis pathways	
	564 Glycerophospholipid metabolism			Deactivation at t7-t8 - ABC transporters,membrane transport system.	
	ABC transporters				
	genes of the secretion system				
**Nucleotide metabolism**		240 Pyrimidine metabolism			
		230 Purine metabolism			
**Replication and repair**	3030 DNA replication	3030 replication			
	3410 Base excision repair	3430 mismatch repair			
	3420 Nucleotide excision repair	3440 homologous recombination			
	3430 Mismatch repair				
	3440 Homologous recombination				
**Transcription/translation**	970 Aminoacyl-tRNA biosynthesis		Ribosomal proteins	Induced in the t2-t4 time interval. RNA polymerase complex genes, ribosomal proteins (L1, L2, L3, L4, L5, L6, L9, L10, L11, L14, L15, L16, L17, L18, L21, L22, L23, L24, L25, L30, S3, S4, S5, S6, S7, S8, S10, S12, S16, S18, S19)	
	ribosomal proteins		970 Aminoacyl-tRNA biosynthesis		

Genes of the TCA cycle followed the general course, being down regulated in the first time interval and reactivated in the second time interval (t2-t3, 0–30 min of growth). As we do not expect decreased demand for carbohydrate metabolism during this period, the initial deactivation was most likely caused by the degradation of mRNAs persevered from dormancy, as discussed above. Considering the centrality of the TCA cycle, it is not surprising that it represented almost half of the carbohydrate metabolism genes that were induced in t2-t3. The second gene reactivation in t7-t8 may correspond to an increase in the demand for energy metabolism possibly due to the initiation of the first replication round and active growth. Genes characteristic of oxidative phosphorylation encode NADH dehydrogenase and ATP synthase subunits. However, unexpectedly the expression of NADH dehydrogenase subunits was repressed during the t7-t8 time interval. Since germination is a very energy demanding process, NADH dehydrogenase and ATP synthase mRNAs were either already present in dormant spores or constitutively expressed in the following periods.

Genes of Lipid metabolism were activated after the initiation of germination (t1-t2) and included mostly genes of Fatty acid biosynthesis pathways (nos.61 and 71), namely, acetyl-CoA acetyltransferase (SCO4921, 6271) and acyl-CoA carboxylase complex (SCO6731, 6788). Curiously, there are two exceptions - acetyl CoA carboxylase and acetyltransferase (SCO2445, 5399) that were down regulated at t1-t2.

Our previous work highlighted the crucial role of the chaperones DnaK, GroEL and Trigger factor in the refolding of aggregated proteins during the initiation of germination [24]. Here, we also show that the expression of DnaK, a member of folding, sorting and degradation group, is enhanced after the initiation of germination (t1-t2 interval). Interestingly, the activation of chaperones was accompanied by the activation of preprotein translocase, which is a membrane transporter for secretory proteins [25]. This indicates the activity of secretory pathways, wherein these particular chaperones could be involved to guard the transport of preproteins. As could be seen from Table 1, the genes corresponding to pathways 310 (Lysine degradation), 350 (Tyrosine metabolism), 360 (Phenylalanine metabolism), and 380 (Tryptophan metabolism) that belongs to the amino acid metabolism group, were not found to be activated in the t7-t8 interval. Tyr, Phe and Trp are aromatic amino acids, being produced by the Shikimate pathway [26]. This suggests that the expression of the Shikimate pathway is coordinated during germination and is mainly expressed during the t2-t3 period.

Opposed to other metabolic groups, transcription-associated pathways were not deactivated in any time interval during germination. The expression of genes involved in the transcription machinery was induced in the t2-t4 time interval, when most mRNAs from dormancy have been degraded and new RNAs and proteins have to be synthesized. The most activated were the RNA polymerase complex genes.

In contrast to transcription, translation-related pathways, including aminoacyl-tRNA biosynthesis pathway and some ribosomal proteins, were down regulated during spore rehydration (t1-t2). This suggests that dormant spores are equipped with all the components needed to restart active protein synthesis. The translation-related pathways, as the transcription-related pathways, were also induced in the t2-t4 time interval. During this time interval, the expression of genes encoding a number of ribosomal proteins (L1, L2, L3, L4, L5, L6, L9, L10, L11, L14, L15, L16, L17, L18, L21, L22, L23, L24, L25, L30, S3, S4, S5, S6, S7, S8, S10, S12, S16, S18, S19) was activated. Ribosomal proteins together with aminoacyl-tRNA synthetase raised again in the 150–180 min interval (t7-t8). During this time interval the initiation of the first DNA replication is expected. This indicates that germinating cells adjust the number of ribosomes and thus protein synthesis to the increase of metabolic activity linked to active growth.

The expression of most of the functional groups was diminished during the first period (t1-t2). In fact, as a dormant spore is metabolically inactive, the time point t1 (dormant) corresponds to the total amount of mRNA synthesized during spore formation stably maintained during dormancy. We speculate that a maturing spore is most likely “not able to predict” the future conditions under which the germination will start. Its transcriptome should potentially be programmed to cover the basic requirements to initiate growth from the storage components present in spores. When germination starts, the expression of the genes required to adjust to the specific environmental conditions is triggered. Other resting mRNAs that are not required are degraded. With this in mind, it is not surprising that the expression of most genes was diminished in the subsequent period t2-t3. Over the course of germination, the number of genes with diminished expression decreased and fluctuated around just a few genes. Exceptions to this rule were the “membrane transport” (mainly ABC transporters) and “signal transduction” groups, which followed the characteristic profile of the genes with enhanced expression, i.e., with two maxima between time points t2-t3 and t7-t8.

### Sigma factors

**Figure 6:**
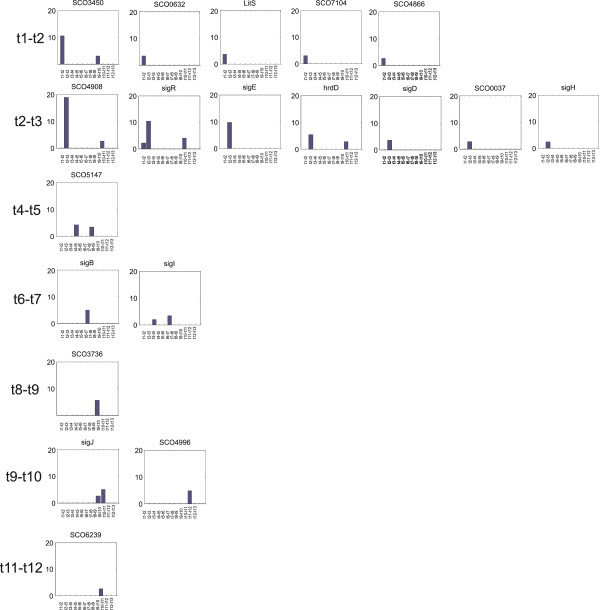
**Differential expression profiles of sigma factors induced during germination.** Sigma factors are sorted according to the time point in which their expression was enhanced (top – down) and the fold-increase in expression level between consecutive time points (right – left, vertical axis). Only intervals with significant changes are displayed.

Strakova et al., showed which proteins were highly expressed during germination [20, 21]; the analysis presented here shows the changes in expression. Interestingly, the latter approach revealed several regulators (such as sigma factors), which do not need to be expressed at high levels but must be active at the exact time when their action is required. The sigma subunits of RNA polymerase are regulatory elements that dictate which genes will be transcribed. Here, 45 genes annotated as sigma factors were analyzed for differential expression between two consecutive time points. Of the 45 sigma factors, expression of 18 was significantly and at least twofold enhanced between at least two consecutive time points. The differential expression profiles of these genes are shown in Figure 6.

Most of the sigma factors were activated in the first phase of germination between time points t1-t2 (the initial 10 min activation period) and t2-t3 (0-30 min of cultivation), including the known sigma factor SigR, SigE, HrdD, SigD and SigH. The highest enhancement in the t2-t3 period was observed for the expression of SigR (10.3×) and SigE (9.7×), whereas HrdD and SigD were induced 5.4× and 3×, respectively, in the same period. This is in agreement with the fact that the cells enter into a metabolically and morphologically different type.

In the t1-t2 time interval, the expression of four other sigma factors with unknown function (SCO0632, SCO3450, SCO4866, SCO7104) was also greatly enhanced, more than 10-fold for SCO3450, and more than 3-fold for the others. The target genes of these sigma factors are unknown. However, since SCO3450 bears 34.1% identity with SigR (discussed below), we propose that this factor controls protein aggregation during the spore activation, as previously suggested for SigR. Although its expression was significantly diminished (3.6×) in the t3-t4 interval, the degree of SCO3450 down regulation was much lower than that of its up regulation in the first interval, indicating an adjustment of its level to a constant value necessary for subsequent growth.

The sigma factor SCO0632, whose expression was also enhanced in the t1-t2 interval, possesses 62.4% identity with SigW of *Bacillus subtilis* [27] that is involved in the response to cell wall damaging treatments, including the degradation by active hydrolases. The activation of SCO0632 at t1-t2 (3.5×) and its down regulation at the following interval (2.4.×) are of comparable magnitude, indicating that this gene is switched off after 30 min of growth.

Another gene, SCO0194/LitS [28], was up regulated in t1-t2 (3.6×) but also to relatively high levels (3.2×) between time points t4-t5. LitS is an ECF sigma factor, whose expression is induced by light. This factor directs the expression of the carotenoid biosynthesis gene cluster. Carotenoids scavenge free radicals and thus protect cells from photo-oxidative damage [29]. As the germination experiment took place in daylight, the reactivation of LitS synthesis may suggest that the spore coats are disrupted, thereby allowing light to enter the cell. Indeed, spores lose their refractibility at the stage of LitS activation, as clearly seen with light microscopy.

### SigE

Streptomycete cell wall anchor CseC sensor and CseB response regulator constitute a signal transduction system responding to various cell wall damages. So its activity is induced by cell wall-specific antibiotics or by the cell wall hydrolytic enzyme lysozyme [30, 31]. The CseB-C two-component system controls the transcription of SigE. Both *sigE* and *cseB* mutants exhibit an identical phenotype that is manifested by cell envelope defects [32]. The cell wall weakness is caused by breaks introduced by internal cell wall hydrolases that are involved in reconstructing the growing cell envelope from the rigid and robust spore coat. Therefore, these hydrolases are mostly active during the initial stages of germination [10, 21]. Our measurements revealed the sequential up regulation of three of these hydrolases: SwlC was induced 3.5× at t3-t4, RpfA was induced 5.6× at t6-t7, and SwlA was induced 5.4× at t8-t9. We think that as a means of recovering the destructive role of these hydrolases, the ECF member SigE (and also possibly the SigW-like SCO0632) is activated in advance to govern their regulon and to manage the process of cell wall reconstruction.

### SigR

The minimal water content of dormant *Streptomyces* spores causes proteins to aggregate. However, as it was shown previously [33], the protein system regains full functionality during germination. The process of protein refolding is controlled by the activity of molecular chaperones, which are abundant and massively expressed during the initial stages of germination. As an example found here, the expression of chaperone DnaK was enhanced (2.2 ×) at t2-t3.

Other stresses such as disulfide stress also leads to protein aggregation. This results in the activation of the SigR regulon, which comprises the DnaK (enhanced at t2-t3 and t7-t8; diminished at t6-t7) and ClpB chaperones, among others. The expression of the latter was moderately but constitutively enhanced mostly during the first part of germination (t1-t2 2.2×, t2-t3 2.3×, t4-t5 2.5×, t9-t10 2.8×).

In *Streptomyces* coelicolor, the ECF factor SigR is known to be responsible for regulating thiol-disulfide redox balance [34]. The involvement of the SigR regulon in protein quality control is documented by the decreased ability of SigR mutants to resolubilize protein aggregates. Interestingly, both the expression of SigR and its anti-sigma factor RsrA was enhanced 10 fold during the same t2-t3 period. The activation of the SigR regulon was represented here by members of the protein degradation pathway, ribosome compounds and chaperones, which were activated at the same t2-t3 interval. This suggests that, although expressed, RsrA does not trap SigR at the t2-t3 period. Since RsrA releases SigR under thiol-oxidative stress conditions [35], we may speculate that the initial phase of germination, being a transition from a dormant to a metabolically active state under aerobic conditions, generates oxidative stress. Following its induction at t2-t3, the expression of SigR was diminished at t6-t7 (4.3×).

### SigQ

The absolutely highest increase (>18×) in the t2-t3 time interval was that of SCO4908, whose mRNA was already abundant in dormant spores (t0). Its expression was temporarily diminished (7.4×) in the rehydration period (t1-t2). The SCO4908 encodes a putative sigma factor, SigQ that is located upstream of the afsQ1-Q2 system. SigQ is known to antagonize the ability of the AfsQ1-Q2 system to stimulate antibiotic production [36]. The hyperinduction of SigQ observed in the present study may thus suggest the SigQ-mediated negative control of the pathway-specific activator genes actII-ORF4, redD, and cdaR during germination and early vegetative growth.

### SigH

SigH showed similar pattern of expression as SigQ. Its expression was also diminished fivefold at t1-t2, compared with its level in dormant spores, and then reactivated in the t2-t3 interval (2.5×). Afterwards, its expression did not change significantly until the end of germination. SigH was suggested to control both the septation of aerial hyphae and, as SigR, the osmotic stress response [33–40]. The osmotic stress response is possibly achieved in cooperation with the anti-anti-sigma factor BldG [41]. The *bldG* mRNA was highly abundant in dormant spores and as well as SigH, its level decreased in t1-t2 interval (4×) and remained constant until the end. On contrary, the re-activation of SigH expression in t2-t3 suggests that its regulon is not only expressed during sporulation but also in germination, when massive water entry likely generates osmotic stress.

### Later activated sigma factors

Expression of the other sigma factors in later periods was not as high as in the first 30 minutes, with only one or two sigma factors activated at each time interval (see Figure 4). The expression of SigJ (SCO1276) and SCO6239 was diminished at t7-t8 (SigJ 4.6×, SCO6239 7.6×) prior to their activation at t9-t10. No other genes from the group of late-induced sigma factors were significantly down regulated during germination. The ECF factor SigJ is thought to be a member of the streptomycete osmotic sensory system [42], as other sigma factors (SigB, SigI, SigH, SigK, SigL, SigM) [33]. In the t6-t7 period, two sigma factors from the osmotic sensory group, SigB and SigI, were activated, indicating their possible role in the subsequent activation of metabolic pathways at t7-t8. In addition to responses to both osmotic and oxidative stresses, the SigB regulon also contains other sigma factors, oxidative defense proteins, and chaperones that play a role in cell differentiation [43]. Since the expression of only a few sigma factors was down regulated during the period preceding t7-t8, the steady state level of expression of the others (including that of SigB and SigI) might control the metabolic processes occurring at t7-t8.

### Sigma factors summary

The sigma factors exhibited a different trend in comparison with the metabolic pathway genes. Whereas most the metabolic pathways were activated between 0–30 minutes (t2-t3) of growth and again between 150–180 minutes (t7-t8), the sigma factors were already up regulated at the beginning of growth (during the 10 min heat spore activation, t1-t2) and, as with the metabolic genes, in the 0–30 minutes interval (t2-t3). These data correlates with their regulatory role in subsequent gene expression. Reactivation after 150–180 minutes (t7-t8), which was noticed for genes of the metabolic pathway, did not occur for most sigma factors.

Sigma factors of the extracytoplasmic function family (ECF) regulate the expression of genes associated with the ability to respond to environmental changes. Streptomycetes ECF sigma factors are known to respond to various stresses to which soil dwelling bacteria are naturally exposed. However, our expression data show that these factors are physiologically activated during developmental processes such as germination. Also other similarities could be found between germination and stress conditions. The conditions that prompt a spore to germinate involve heat shock (used in our experiments to induce synchronous germination), osmotic as well as oxidative/disulfide stresses. Activation of SigR, SigH and LitS signals oxidative stress as that of SigB, SigH, SigI, SigJ signals osmotic stress. Protein quality control is under the control of SigR and possibly SCO3450. Cell wall damage responses are also activated during germination since this process requires the massive reconstruction of the cell envelope. The sigma factors responsible for cell wall reconstruction are SigE and possibly SCO0632, which is homologous to SigW of *Bacillus*.

## Conclusions

Differential expression analyses have provided a new view of the germination of streptomycete spores. As shown here, dormant spores carry not only DNA itself but also an ample transcriptome. Considering the minimal metabolic activity of dormant spores, most of mRNAs required for the initial phases of germination must be supplied during sporulation. After rehydration of the spore, mRNAs are being degraded in the t1-t2 period and the synthesis of mRNAs is delayed. The decreased stability of RNA molecules could also be partially caused by the increased temperature (50°C) during the initial 10 min of cultivation, which led to the synchronous spore activation [33].

Alternatively, active intracellular RNases might be involved in the initial degradation of mRNAs. In contrast, the expression of a small portion of genes is maximally activated at this stage, including several components of lipid metabolism (for membrane synthesis) and the membrane transport pathways.

Those mRNAs that are required for further development are re-synthesized mostly during the subsequent t2-t3 period or later. This increases the need for elements of the transcriptional apparatus in t2-t3, whose own mRNA/rRNA/tRNA pool seems to have escaped the initial RNA degradation, thus making it an exception. The transcription and translation apparatus is activated continuously throughout the two initial periods t1-t3.

Most of the metabolic pathways remained active at a basal level throughout the first 150 min of growth, when next burst of metabolic activity in t7-t8 occurred. We believe that this burst is necessary to initiate the first round of DNA replication. After the period of 150–180 min (t7-t8), the activity of most of the pathways remained constant until the end of the measured period (5.5 hours). Our study demonstrated that germination requires the concerted action of a number of sigma factors, whose expression is also activated from the beginning: the putative sigma factors SCO0632 (SigW-like), SCO3450 (similar to SigR), and LitS at t1-t2 and the activation of HrdD and the known ECF family sigma factors SigR, SigE, SigD and SigH in the t2-t3 period. However, the highest activation at this stage belonged to SigQ (SCO4908), whose function in germination is unknown. SigR, and most likely SCO3450, ensure quality control in the re-folding of proteins that aggregated in dormancy and are required during germination. SigE, and most likely SCO0632, control cell wall integrity, possibly because a massive reconstruction of the cell envelope is needed after the developmentally regulated destruction of the spore coat by the cell wall hydrolases. Based on the induction of other sigma factors, most of which are known to respond to various stresses, we suggest that germination evokes stress-like responses as a consequence of the disruption of the internal steady conditions of the spore, which leads to an influx of water, oxygen (and its radicals), salts, and even light, as reflected in LitS expression.

## Methods

### Cultivation and germination

Details regarding the cultivation and growth of *S. coelicolor* M145 spores were published in our previous work [20, 21, 33]. Briefly, sporulation proceeded 14 days at 28°C on solid agar plates (0.4% (w/v) yeast extract, 1% (w/v) malt extract, 0.4% (w/v) glucose, 2.5% (w/v) bacterial agar, pH 7.2) overlaid with cellophane discs. The harvested spores (sample t1 taken after growth for 14 d) were first activated by mechanical disruption of the outer coat in water and then washed, and a 10 min heat shock treatment (50°C in AM medium, sample t2 taken) was applied to boost synchrony. Spores were then germinated in liquid AM medium (20 amino acids at 0.2 mM, 20 mM KH_2_PO_4_, 30 mM Na_2_HPO_4_, 2% (w/v) glucose, 0.05% (w/v) MgCl_2_, 0.5 mM CaCl_2_, 7 mM KCl, and a mixture of bases, each 0.01% (w/v)) at 37°C (samples t3 – t13 taken every 30 min). mRNA samples were collected from dormant (t1) and heat-activated (t2) spores and then at 30-min intervals throughout the germination time-course until 5.5 h (t13) of growth. Altogether, we obtained samples at 13 time points, including samples from dormant spores, with at least three biological and technical replicates for each time point.

### RNA isolation from spores

To disrupt the cells, we used a FastPrep-24 machine (Biomedicals), wherein the spores were mechanically shaken in tubes containing zirconium sand, two 4-mm glass beads, 500 μl of lysis buffer [44] (50 mM Tris–HCl pH8, 500 mM LiCl, 50 mM EDTA pH 8, and 5% (w/v) SDS) and 8 μl of RNase inhibitors (Bio-Rad). The samples were centrifuged at 14000 g for 15 min at 4°C, and phenol-chloroform RNA extractions were performed twice on the supernatant. The RNA was precipitated overnight in ethanol and 0.3 M sodium acetate at −20°C. Finally, the RNA was re-suspended in 50 μl RNase-free water and 0.5 μl RNase inhibitors, and the remaining DNA was removed using a DNase kit (Ambion). The RNA was stored in water at −20°C.

### DNA microarrays and data processing

Data were processed as in our previous paper [21]. The data preprocessing steps are repeated here to make clear how the values used for the analysis in this article were obtained.

RNA quality control and gene expression levels were performed by Oxford Gene Technology (Oxford, UK) using Agilent DNA microarrays covering the entire *S. coelicolor* genome and the standard Bacterial RNA amplification protocol for two-channel assays by OGT.

The data were normalized using LOWESS and filtered for background and flag information (from Agilent documentation) in the GeneSpring software to obtain genes that were expressed significantly above background and to avoid side effects of possible cross hybridization. These methods reduced the number of entities on a single array from 43888 to 25312, which finally represented the outcome for 7115 genes out of 7825. The data discussed in this publication have been deposited in the NCBI Gene Expression Omnibus [45] and are accessible using the GEO Series accession number GSE44415 (http://www.ncbi.nlm.nih.gov/geo/query/acc.cgi?acc=GSE44415).

### Array normalization

The experiment included 37 arrays from 13 distinct time points during *S. coelicolor* germination. The arrays shared a common reference in the red channel (Cy5), which consisted of a mixture of RNA samples from all examined time points. The distributions of Log2Ratio values (Log2Ratio = log2 (Sample (Cy3)/Reference (Cy5))) for all samples were scattered around a common mean and all had similar variance. Therefore, the distributions for each array were centered so that the medians and the median absolute deviations of all the array distributions were equal. To eliminate array outliers, we filtered out the 0.02 quantile of the least and the most intensive Log2Ratio values. Normalized Log2Ratios were exponentiated to return the values to the original scale further refered as the Normalized Ratios.

The outliers among gene replicates at individual time points were filtered using the Q-test (for 3–9 inputs) and the Pierce test (for > 10 inputs).

### Expression profile analysis

#### Highly expressed genes

To eliminate profiles with low overall expression during germination, we analyzed microarray sample channel signals (Cy3 labeling). The idea was to minimize the influence of gene profiles whose microarray signal originated from experimental errors that exceeded the pure technical limits for eliminating signals under the background. Thus, the overall expression level for each gene was specified by computing the median across all microarray replicates at all time points for the sample channel microarray signal. Profiles whose overall expression level was below the first quartile value (563) of all counted medians were filtered out. To avoid omitting profiles with a low overall expression level but with a significant peak, the filtered expression profiles were manually inspected; in the presence of a significant peak, the profile was considered to be highly expressed and added to the set. The final set of “highly expressed” genes contained 5385 gene expression profiles and was used for further analyses.

### Differential expression analysis

Each gene in the “highly expressed” set was represented at each time point by a set of Normalized Ratios corresponding to the array and sample repeats. At each time point, the Normalized Ratios for a single gene were distributed around a mean with a variance given by the experimental and biological error. The mean of the Normalized Ratios at a given time point will be mentioned as the “gene expression level” of the given gene at the given time point t (mRNA_i_t_j_, i = 1..5385, j = 1..13). A series of expression levels at all 13 time points will be further mentioned as the “expression profile”. The differential expression of gene i between two consecutive time points, t_j_, t_j+1_, was then calculated as the ratio of the expression levels of the given gene at the two consecutive time points (mRNA_i_t_j+1_/mRNA_i_t_j_) for all 13 time points (j = 1..12), resulting in a “differential expression profile”. The significance of the difference between the expression levels at t_j_, t_j + 1_ was tested by a standard t-test for equality of means. At each interval (t_j_, t_j+1_), genes with significant differences in their levels (p < 0.05) and with an activation/deactivation ratio >2 were selected (the 2-fold change level was arbitrarily chosen to exclude significant but biologically irrelevant changes in expression levels). All genes with significant change in expression and greater than 2-fold level at at least one interval during germination were identified in the KEGG database (Kyoto Encyclopedia of Genes and Genomes, http://www.genome.jp/kegg/) [22] and mapped to pathways. The same procedure (without KEGG mapping) was also carried out for 45 annotated sigma factors. Temporal changes in the differential expression profiles were analyzed, and the results were summarized.

## Electronic supplementary material

Additional file 1: **Activation/deactivation of genes between two consecutive time points.** (XLSX 6 MB)

Additional file 2: **Movie representing activation of metabolic pathways during the course of germination depicted in the KEGG base pathway map.** (MP4 16 MB)
